# Cytokine Receptors in Development, Homeostasis and Disease

**DOI:** 10.3390/ijms241210352

**Published:** 2023-06-19

**Authors:** Alister C. Ward

**Affiliations:** 1 School of Medicine, Deakin University, Geelong, VIC 3216, Australia; alister.ward@deakin.edu.au; 2 Institute for Mental and Physical Health and Clinical Translation, Deakin University, Geelong, VIC 3216, Australia

This Special Issue represents a collective celebration of the cytokine receptor superfamily and the myriad of functions mediated by these important molecules in development and homeostasis, as well as their disruption in disease. The correct development and ongoing maintenance of specific cell lineages are essential underpinnings of a healthy organism, with their perturbation directly associated with numerous diseases. The mechanisms regulating these processes are many and complex, with cell–cell communication being pivotal. Cytokine receptors are located on the exterior of responsive cells, where binding by cognate cytokine molecules leads to the transmission of signals into the interior of the cell to impact various critical functions. In this manner, signaling via cytokine receptors regulates the normal development and function of multiple cell types, particularly blood and immune cells, with many pathological states associated with the disruption of cytokine receptor signaling.

The cytokine receptor superfamily is broad. The type I family contains conserved extracellular motifs and includes receptors for many interleukins (ILs), colony-stimulating factors (CSFs) and other cytokines, while the related type II family acts as receptors for interferons (IFNs) and other ILs. Both of these families signal through the important Janus kinase/signal transducer and activator of the transcription (JAK/STAT) pathway to mediate their effects. The tumor necrosis factor (TNF) receptor family has a common cysteine-rich extracellular binding domain and both cytokine and non-cytokine ligands. The transforming growth factor-beta (TGFβ) receptor family consists of serine/threonine kinase receptors, while the large chemokine receptor family comprises G-protein-coupled receptors. Finally, some members of the immunoglobulin (Ig) family act as receptors for cytokines, including IL-1 and CSF-1.

This Special Issue encompasses a collection of interesting original research articles and topical reviews that explore aspects of cytokine receptor biology and related pathology. Several contributions focus on type I cytokine receptors. The first is an article that details the generation and characterization of a zebrafish mutant in the common IL-2 receptor chain, IL-2Rγc, based on those observed in human severe combined immunodeficiency disorder (SCID) [1]. The mutants exhibited a significant decrease in both T and NK cells with defective tumor immunity and a dysregulated microbiome, validating them as a bone fide SCID model. The second contribution is a review that describes the pleiotropic action of oncostatin M/gp130 in cardiomyocytes—specifically, how acute activation protects the heart following injury, whereas chronic activation promotes heart failure [2]. The third contribution then reviews the roles of two IL-6 family cytokines, cardiotrophin-like cytokine factor (CLCF) 1 and cytokine receptor-like factor (CRLF) 1, across both development and pathological conditions, notably including cold-induced sweating [3]. Two other contributions place a spotlight on TNF receptors (TNFRs). This includes an article that investigates peptides derived from the innate immune protein Tag7 and their ability to activate TNFR1 (and another cell surface molecule, TREM-1) to inhibit pro-inflammatory cytokine production [4], and a review of the interaction between TNF, TNFR1 and TNFR2 in bone-related cells [5]. The final contribution is a review that examines the role of a broad set of cytokine receptors in immunity against mycobacteria, including examples of type I, type II, TNF, TGFβ, chemokine and Ig receptors [6]. It is hoped that this Special Issue places a renewed focus on the fundamental importance of cytokine receptors, furthering our understanding of their role and potential as therapeutic targets in relevant diseases.
